# Viewpoint: Finance needs of the agricultural midstream^☆^

**DOI:** 10.1016/j.foodpol.2023.102530

**Published:** 2023-11

**Authors:** Kate Ambler, Alan de Brauw, Sylvan Herskowitz, Cristhian Pulido

**Affiliations:** a International Food Policy Research Institute, United States; b World Bank, United States; c Innovations for Poverty Action, Colombia

## Abstract

Recent literature suggests that agricultural value chains are changing rapidly and places an increasing focus on the importance of actors and activities taking place in the “midstream” of these value chains, after production and prior to final sale. This article discusses the financial needs of midstream actors in agricultural value chains, emphasizing differences across midstream activities and highlighting how value chain characteristics can influence both financial needs and potential remedies. The paper concludes with a brief discussion of the prospects of digital financial services and policy levers for government actors in this space.

## Highlights

• The agricultural midstream is relatively understudied.
• The vast majority of firms in the midstream are MSMEs in potential need of finance.
• Needs are heterogeneous by agricultural conditions, seasonality, and policy environment.

## 1 Background and motivation

Agricultural economics research in developing countries has traditionally been centered on smallholder farmers and on-farm production. Smallholders constitute a majority of the world’s poor, and a long-standing (though debated) view in development economics suggests that increasing smallholder productivity is essential for poverty reduction (e.g. Johnston and Mellor, 1961) and possibly for broader economic growth as well (Timmer, 2002).^1^ The central role of smallholder farmers was reinforced by the 2008 World Development Report and the concurrent food price crisis (World Bank, 2007), catalyzing a renewed focus on international aid directed towards agriculture. Despite these more recent efforts and generally strong economic growth throughout the developing world prior to the COVID-19 pandemic, rural poverty remains a persistent challenge (UN, 2022). The persistence of poverty among smallholders and the large gaps between prices paid to producers and those paid by final consumers suggest that thinking more holistically about rent distribution along agricultural value chains could lead to improved outcomes among both smallholders and midstream value chain actors in low and middle income countries (LMICs; e.g., Yi et al., 2021, de Brauw and Bulte, 2021, Bellemare et al., 2022). Ultimately, for agricultural development to become a more realistic policy objective and effective poverty reduction tool, a deeper understanding of the behaviors, constraints, and interlinkages of value chain actors is needed.

A growing body of research suggests that the composition, roles, and services taking place in the agricultural sector are rapidly changing (Barrett et al., 2022). These changes are particularly pronounced in the “midstream” of agricultural value chains, encompassing activities that occur after on-farm production and before retail. This evolution is simultaneously leading to emergent opportunities as well as new challenges (Reardon, 2015).

The overall scale of the agricultural midstream is difficult to quantify. Dorosh and Thurlow (2018) use a dynamic computable general equilibrium model to estimate the share of the manufacturing sector that is agro-processing in five African countries, finding it represents between 27 and 64 percent of manufacturing. Yet this method leaves out any value added beyond packaging (for example, meals at restaurants) as well as transportation. In a companion paper to this one, we use Living Standards and Measurement Study (LSMS) and World Bank Enterprise Survey (WBES) data from seven African countries to identify and describe household enterprises and firms engaged in midstream agricultural activities (Ambler et al., 2022a). The LSMS data suggest that at least 16% of household enterprises were engaged in what could be considered midstream value chain activities with 31% of those in the *agricultural* midstream. In the WBES data, we find that 52% of firms were engaged in midstream activities with 17% of those in the agricultural midstream. However, given the challenges of identifying the activities and industries of businesses in those two data sources, these figures may constitute lower bounds of their overall incidence.^2^ Still, the research base on these actors and their activities remains quite limited (Bellemare et al., 2022, Barrett et al., 2022).

While limited, this literature suggests that a large share of activity in the agricultural midstream is conducted by micro, small, and medium sized enterprises (MSMEs; Reardon et al., 2021). The productivity growth of these enterprises could be hampered by several different factors, including poor infrastructure, a lack of appropriate skilled labor, and poor contract enforcement, among others (e.g. Fafchamps and Minten, 2001, Swinnen and Kuijpers, 2019, Barrett et al., 2022). Yet one challenge that appears overlooked in the agricultural value chain literature is the ability of midstream firms to access financial services to expand their businesses. A review of surveys conducted among midstream actors in agricultural value chains found that details of financial relationships or transactions were not the focus of most data collection exercises. Only half of surveys reviewed asked about access to credit or the presence of formal contracting, and questions about transaction details (loan amounts, interest rates, terms) were less frequent. Other finance related questions, including risk mitigation, savings, and payment methods were rarely asked (Ambler et al., 2022b).^3^

Wherever new economic opportunities emerge, financial needs along with the availability of suitable financial services play a critical role in both facilitating investments and determining who is (and who is not) able to take advantage of them. Access to credit, secure saving, and reduced exposure to risk can facilitate investments, while low-cost modes of payment and market coordination services have the potential to reduce transaction costs, creating greater surplus and potential efficiency gains. While we know little about the specific financial needs of MSMEs operating in agricultural value chains, some reports suggest they are large; for example, excluding India, ISF Advisors (2022) estimate that financial needs for investment among agricultural SMEs in Africa and Southeast Asia may be as large as $160 billion. The wide variety of agricultural contexts, agroecological conditions, and policy environments facing value chain actors may further shape opportunities available in specific agricultural value chains and help or hinder whether different financial products can be offered within the agricultural sector.

Midstream actors in agricultural value chains are likely to face a wide array of constraints shaping their decision-making processes. They face labor, infrastructure, and environmental challenges, and many operate in settings with weak property rights and contract enforcement. Financial capabilities and needs interact and are intertwined with these other challenges. For example, labor or infrastructure constraints can affect the financial options or even needs that a firm has, changing the returns to additional access to finance. On the other hand, constrained access to finance can also affect those constraints; firms, for example, might not be able to search for the right types of labor to overcome labor constraints without access to finance. While recognizing that midstream actors face multiple constraints and it is not possible to necessarily overcome financial constraints without addressing or at least recognizing these other constraints, we focus this paper on midstream finance both to narrow its overall scope and in recognition that financial needs and constraints in the midstream of agricultural value chains is an area in need of increased research attention. While extensive research analyzes financial constraints among smallholders, research on financial needs among midstream actors is relatively sparse.

This paper is an effort to give focused attention on the midstream to broaden our understanding of their financial needs. In addition, the landscape for agricultural finance is changing rapidly and emerging digital financial services (DFS) have the potential to meet needs of midstream actors that are either unmet by existing policies and the formal financial sector or costly when provided through the informal sector. DFS adoption among midstream actors may help improve financial inclusion among smallholders who have been difficult to reach. However, constraints and low uptake by smallholders may also hinder DFS adoption and benefits among midstream actors, resulting in a low-adoption equilibrium, or exclusion of those in less connected areas.

In this paper, we consider the financial needs of midstream actors working within agricultural value chains. We first provide a broad categorization of midstream actors, sketching their main activities and financial needs. We then detail a set of important value chain features along with a brief discussion of their implications for financial needs of midstream actors. We then discuss the policy environment for midstream financial services, followed by a review of how midstream financial needs are currently being addressed. We conclude with directions for future research on finance and the agricultural midstream.

## 2 The midstream and its financial constraints

Recent research has highlighted several important recent changes in agricultural value chains in developing countries. Important drivers of these changes include rising income levels, changing relative prices for different food types, urbanization, changing demographics, shifts in trade patterns, and evolving technology (Reardon et al., 2012, AGRA, 2019, Barrett et al., 2022). Acceleration of these factors has led to assertions that rapid midstream growth is creating new economic opportunities (Minten et al., 2014, Reardon, 2015, Hernandez et al., 2018), employment (Yeboah and Jayne, 2018), and rural income-generating activities (Dolislager et al., 2021, Reardon and Minten, 2021). Beyond income generation for midstream actors themselves, the functioning of the midstream has implications that extend to the ends of the value chain, affecting both the incomes of small-scale producers and the budgets and nutritional status of consumers (Liverpool-Tasie et al., 2020, Yi et al., 2021). As such, understanding the financial constraints, risks, and opportunities faced by midstream actors is key for developing policy that will help allow the midstream to meet its full potential. In this section, we describe classes of actors within the midstream and the financial needs they face.

The composition of midstream activities depends on the country context and agricultural product, but typically includes transport, trading and wholesaling, processing and packaging, and storage. Businesses in the midstream can take on a wide variety of forms, from formal to informal, and while businesses often center on a specific midstream activity, some midstream actors may perform more than one value chain role. For some products, agricultural producers or producer groups may do some activities themselves, particularly related to value addition or preservation, before sale. In some contexts, services such as transport or storage may be provided across value chains or even across sectors (Reardon, 2015).

The specific geographic context and agricultural product matter greatly, reflected in different value chain structures, activities, and participants, making broad generalizations difficult. Regardless, we offer a stylized characterization of the main activities based on a range of academic and gray literature. Table 1 summarizes these core activities, providing their source of profits, phase of the value chain, long-run credit and short-run liquidity needs, risk exposure, and other financial considerations.

**Table 1:** Midstream Activities.

**Activity**	**Source of Profits**	**Phase of Value Chain**	**Long-Run (LR) Credit and** **Short-Run (SR) Liquidity Needs**	**Risk Exposure**	**Other Financial Considerations**
**Transportation**	Spatial arbitrage	Aggregation and Distribution	SR: Liquidity for purchase of commodities from producers LR: Purchase of transport equipment	1. Damage to capital from bad infrastructure 2. Uncertain supply 3. Theft of cash	1. Search costs 2. Principal-agent misalignment of incentives
**Trading and Wholesaling**	Price mark-ups	Aggregation and Distribution	SR/LR: Rent or buy commercial space LR: Credit to purchase produce post-harvest at low prices, repay over time/later when prices rise	1. Buyer and seller price risk 2. Spoilage 3. Uncertain supply/demand 4. Theft 5. Side selling	1. Delivery commitment 2. Transaction costs 3. Liquidity needs
**Processing and Packaging**	Value addition	Value Addition	SR: Liquidity needs to pay for labor LR: Investment in processing/packaging equipment	1. Irregular supply 2. Low quality supply 3. Uncertain input prices 4. Uncertain global prices	1. Domestic/ international ownership 2. Export orientation
**Storage**	Inter-temporal arbitrage; Value preservation	All	LR: Investment in storage facilities LR: Capital to purchase goods while waiting for arbitrage opportunity (or timing of production)	1. Spoilage 2. Pests 3. Theft 4. Uncertain long-term prices 5. Electricity supply (cold storage)	1. Delivery commitment 2. Transaction costs 3. Liquidity needs

Trading and wholesaling are frequently linked with transportation services. In many cases, traders expend energy identifying sellers and use their own vehicles to arrange transport (Fafchamps, 2003). After accumulating sufficient volumes, larger traders may take the form of wholesalers who establish their presence in urban centers or rotate through local markets, letting nearby farmers bring their produce to them. Both frequently play an important role in aggregation of products as they move towards processors, exporters, or in preparation for within-country distribution to local consumer markets. Trading between farm and wholesalers can take many different forms; for example, maize is traded all over Africa, and can be initially transported away from small farms on anything from a bicycle to an ox-cart to a truck; wholesalers may have their own space or rent it from regional markets.

Both trading and wholesaling require short-term liquidity to facilitate high transaction volumes, and improved access to short-term credit can assist small traders in buying larger quantities and growing their businesses. Longer-run credit needs may be required to acquire retail space or to accommodate the seasonality of buying large quantities at harvest and recouping the investment later in the year. At the same time, financial commitments are complicated by the price risk faced by traders and wholesalers. Accessing credit based on the price a trader pays to a farmer can lead to default if the trader faces dropping prices before sale of the product is possible.

Processing activities include any activities occurring post-harvest that add value to a product. In the context of midstream activities, milling grains, drying coffee, treating milk, or more broadly the conversion of raw commodities into saleable or consumable products fall under processing. Some activities face low levels of risk after production and require minimal up-front costs, such as a simple tarp or a mortar and pestle. Small-scale local maize mills may require slightly more capital investment with rural entrepreneurs purchasing gas operated grinders to service the needs of local farmers, while taking on additional uncertainty and risk of theft, damage, breakdowns, and reliance on fluctuating local production and demand for the service. At the top end, large-scale factories may be involved with production of more heavily processed goods, such as biscuits or yogurt that require substantial amounts of capital in the form of industrial mixers, ovens, and conveyors. Maize, for example, is often ground in large mills and blended with soy for chicken feed. These investments typically have high overhead costs making them more vulnerable to risks from unreliable input sourcing or access to labor. Large capital investments require higher production volumes to recoup investments while loss or spoilage can pose additional risks when any part of the production process is out of sync with the rest.

Packaging is needed to preserve the quality of most processed goods. For example, packaging is needed to preserve the freshness of recently produced yogurt, freshly roasted coffee, or canned foods; packaging can also be used to make fresh fruits and vegetables more attractive for the purpose of better marketing and higher retail prices. It can further serve an important role in reducing the risk of damage during transport that would otherwise cut into profits. Returning to our example, larger maize mills will package and brand their maize flour.

Next, storage is important for commodities with highly seasonal production. Wide variation in pre- versus post-harvest prices allow for potential profits from storage or may be conducted by processors themselves who want to ensure a steady supply of inputs needed to meet year-round demand. These activities frequently require access to credit to fund payment of post-harvest prices and accommodate delayed repayment from later sales. Additionally, storage faces risks of spoilage and uncertain later prices which may undercut anticipated profits. Storage occurs across the value chain, by farmers, midstream actors, retailers, and sometimes by government-run warehouses. A variant of conventional storage is cold storage which combines standard storage dynamics with the specific climate requirements for preservation of perishable products. Capital requirements such as refrigerating facilities can be very expensive and require substantial access to credit. Further, cold storage requires a reliable source of electricity, so that outages do not lead to spoilage and lost produce.

In addition to the risks described above, midstream actors face different degrees of price and upstream production risk. While production risk is faced most directly by farmers and producers, risk in the form of inconsistent supply is also transmitted to midstream actors. Processors, for example, often make capital investments for equipment, but depend on a sufficient stream of product from producers to be profitable. Fluctuating prices caused by variable harvests and global price fluctuations can also affect the profits of both farmers and midstream actors. When substantial investments are made anticipating lower prices and greater abundance of product supply, this combination can constitute meaningful risk for these midstream actors. Unexpected reductions in price within season between purchase and sale are also risks for midstream actors. While formal insurance products, such as index insurance, exist in some countries to target production risk for small scale producers, to our knowledge no similar viable product targeting MSME midstream actors exists.^4^ On the other end, global price fluctuations are also rarely covered by insurance products, can move unpredictably, and result in one year’s profitable businesses becoming unprofitable the next.^5^

This categorization serves as a starting point for how to think about some of the main financial constraints present in the midstream of most value chains. While this characterization is intended to anchor initial intuition, the context and structure of a given value chain are critical for anticipating how constraints or financial challenges facing midstream actors, such as a lack of credit or exposure to risk, may affect them and potentially be transferred to other actors at other nodes, both up and down stream.

## 3 Value chain heterogeneity

The previous section outlined how different midstream activities have their own inherent sources of risk and capital or liquidity needs. However, a range of additional value chain characteristics influence these financial needs and options available for meeting them. While many of these contextual factors vary with a value chain’s degree of modernization, others are inherent to the country context or characteristics of a given product.^6^

A handful of recent papers have described this evolution of value chain modernization and delineated three broad stages of development. We reference the main observations here, but recommend referring to those papers themselves for a complete discussion and set of references underpinning these groupings.^7^ Barrett et al. (2022) argue that agricultural value chains in low and middle-income countries are undergoing a “revolution”. This revolution is characterized by a series of stages of modernity where important value chain features vary as they progress from traditional, to transitional, to modern. As value chains modernize, the nature of midstream activities evolves as well. We adapt and expand on their framework to match our categories, summarized in Table 2.

**Table 2:** Midstream Activities by Stage of Value Chain Modernization.

	**Traditional**	**Transitional**	**Modern**
**Transportation and Logistics**	Own transport and logistics by small brokers	SMEs in third party logistics (3PLS)	Large companies and freight forwarders
**Trading and Wholesaling**	Brokers based in rural villages	Wholesalers based in urban markets	Off-market distribution companies
**Processing and Packaging**	None (home-processing)	SMEs such as small mills	Large processors and food manufacturers
**Storage**	In home or village	Central locations with improved facilities	Central locations with improved facilities (incl. cold storage)

As value chains modernize, transport and logistics progress from own transport and small brokers to third party logistics services, and eventually large companies and freight forwarders in modern value chains. Trading and wholesaling progresses from locally-based brokers, to urban-based markets in transitional markets, and off-market distribution companies in modern value chains. This progress is apparent in rice value chains throughout Asia, where transformation of the value chain has led to new businesses, such as mechanized harvesting rental, but has also led to consolidation of mills and more product differentiation through mill-branding for consumers (Reardon et al., 2014). Traditional value chains have little or no processing and packaging, in transitional value chains processing and packaging begins to be done by local MSMEs, and in modern value chains it is largely done by large scale processors and manufacturers. Finally, storage is often done at home or locally in traditional value chains, in central locations for transitional value chains, and in improved and centralized warehouse facilities in modern value chains. Across all these activities, the capital requirements and scope of activities generally expand. This changing nature of activities is both a response to and an enabling factor contributing to value chain evolution. Understanding where a specific value chain is in its development/transition speaks to both the financial needs of the midstream actors and the type of financial solutions that may be appropriate.

We next consider eight characteristics of value chains and how they differ with a value chain’s degree of modernization, summarized in Table 3. These are also adapted from Barrett et al. (2022), making additions to help focus on midstream actors. The first four characteristics relate broadly to the logistical structure of the value chain: value chain length, the end market, spatial dispersion, and the influence of seasonality. The second four relate broadly to market structure and relationships: market power, midstream entry costs, apex actors, and exchange relationships. Below we discuss these characteristics and how they relate to the midstream and their financial needs.

**Table 3:** Value Chain Characteristics by Stages of Modernization.

	**Traditional**	**Transitional**	**Modern**
**Logistical Structure:**			
Value chain length	Short	Medium	Long
Spatial dispersion	Local	Rural-urban	Rural-urban, international
End market	Local	Regional/National	National/International
Influence of seasonality	Very High	High	Medium
**Market Structure:**			
Market power	Local	Local/Regional	Local/Regional/Global
Midstream entry costs	Low	Moderate	High
Apex Actors	None	Some	Prevalent
Exchange relationships	No or informal contracts and few standards	Informal and formalizing contracts, public standards, some vertical integration	Emerging contracts, private standards, vertical integration

These three types of value chains can even be present within the same village (de Brauw and Bulte, 2021). Take, for example, the case of central Malawi, where farmers grow maize, soy, and tobacco, among other crops. Much of the maize sold by farmers is sold through informal channels, so into a traditional value chain. Soy, however, is not part of the traditional diet, so most farmers producing it sell it; the midstream entry costs though are not high as much of the trade ends at producers of poultry feed. This chain can be considered transitional. Some farmers grow tobacco, which is a key export commodity for Malawi and is typically contracted, graded, and sold at a central auction floor to international buyers, so the value chain has clear modern characteristics.

### 3.1 Logistical structure

The logistical structure of a value chain has a major role in determining financial needs of midstream actors as well as shaping the space in which they act. First, value chains can be characterized by their length, or the number of nodes through which an agricultural product passes before reaching the consumer. Length therefore relates directly to the number of midstream activities contained in a value chain. Whereas a short value chain could be as simple as a smallholder walking to a local market and selling their produce to nearby villagers, value chains tend to lengthen as they modernize, passing through a larger number of intermediaries transporting, aggregating, storing, and adding value before reaching final consumers (Barrett et al., 2022). In the former example, with no intermediaries, the price paid by the final consumer is the same as that received by the producer. More typically, each step in a value chain requires its own payments and markup, creating a wedge between prices at farm gate and final vendors. Longer value chains also create wider spans of vertical inter-dependency. While value chain length may not directly determine need for finance or credit, it can influence the type of financial services needed. As we discuss later, vertically-linked value chain actors may influence each others’ financial needs and offer negotiated solutions. Vegetable traders and exporters in Madagascar provide credit for inputs and guaranteed prices to farmers to improve their supply (Minten et al. 2009). However, these arrangements become more complicated when more intermediaries exist between the actors with credit needs and those in the value chain who may have the most resources to help address them.

Next, related to but distinct from value chain length, the extent of a value chain’s spatial dispersion can affect search costs, transportation costs, logistics procedures, and aggregation challenges facing these actors. As value chains modernize, expansive and lengthening chains may introduce greater exposure to specific risks, such as theft of cash, damage to capital, or principal-agent issues between value chain business owners and employees.^8^ Financial services could help mitigate some of these risks by allowing for more secure forms of transactions, improving coordination, and reducing search costs, and insuring or distributing more of these potential risks. As value chains expand over space, financial transaction costs can grow, necessitating modern services to reduce those costs in transitioning value chains (BTCA 2016).^9^

As value chains modernize, they also tend to serve larger and more distant end markets progressing from local markets, to regional, national, and eventually international end markets (Barrett et al., 2022). This tendency is again linked with both chain length and spatial dispersion, but the type of end market can create unique requirements. National and international markets may require different quality standards that both necessitate increased investments and better record keeping, both of which require expanded financial services. Moreover, as midstream enterprises that serve end markets are often large, they likely have better access to finance through formal channels.

Finally, the seasonality of agricultural production and timing of harvest in many value chains have direct implications for the timing of the logistical needs of the value chain. While seasonality typically has the most direct impact on producers, who face highly seasonal credit needs and exposure to risk, this also impacts midstream actors reliant on this production as inputs (Burke et al., 2019). Rain-fed agricultural systems are, of course, more directly affected by seasonality whereas animal products typically have relatively smoother production cycles. While most value chains remain dependent on the natural growing season, creating irrigation systems or greenhouses can extend or add growing seasons to production as value chains modernize.

Most rain-fed agricultural systems have relatively synchronized harvests of primary grains and legumes that can create increased short-term credit needs for midstream actors focused on purchasing, distributing, and processing these products. Where robust warehousing systems do not exist, the greater the volume of a country’s agricultural production that is harvested at or near the same time, the greater will be the seasonal liquidity needs of traders and transporters in the months following harvest. Where possible, some midstream actors may work across multiple value chains with different seasonal patterns so that their capital investments are used at higher capacity throughout the year. This may have an additional benefit to these traders of spreading risk in the case of a bad growing season for a particular crop. These challenges are less relevant in less-seasonal (typically non-rain dependent) value chains, such as milk, eggs, meat, or aquaculture, making financial needs of such firms more closely resemble those of other non-agricultural businesses. Loan products in highly seasonal value chains will need to accommodate the lags between purchase and eventual resale for these midstream actors and where a rush on credit occurs during specific, post-harvest seasons, liquidity may be scarce throughout the entire financial system. More broadly, the availability of suitable financial products for entrants offering feasible repayment schedules and non-prohibitive interest rates are central to competition at these nodes of the value chain.

### 3.2 Market structure

Market structure may have competing effects on value chain function and the financial needs of midstream actors within value chains. In this section we highlight four factors distinguishing different value chains related to their market structure: market power, entry costs, apex actors, and exchange relationships.

First, the distribution of market power at different nodes along a value chain can have a major influence on how value chains function as well as the opportunities for finance. The presence of market power at a given node of a value chain changes the distribution of rents to actors transacting at that node (Fafchamps, 2003). When a buyer has market power, it can affect vertical relationships and arrangements within the value chain, affecting negotiated prices or the distribution of risk in favor of those with greater market power and leverage. As a result, returns to investment among smaller actors can be reduced, which can further reduce banks’ willingness to lend to those actors or the willingness of new potential entrants to invest. However, these larger scale actors may risk exposure among other actors in the chain by providing a guaranteed market for farmers or traders. Without resolution of this output market risk for producers, the midstream actor may never be able to source enough inputs to justify their own investments.

In general, the most competitive market structure in LMICs are domestic grain markets, which typically include the largest number of actors in the midstream and can be thought of as generally competitive, with poor signaling of quality differentiation (e.g. Sitko and Jayne, 2014, Abate et al., 2021). In such markets, contracts tend to be relational and informal, and larger actors often are larger due to larger social networks (Fafchamps and Minten, 2002). As these markets begin to modernize, value chains transform and the structure of the midstream can change. For example, Reardon et al. (2014) show that as several countries in Asia have grown and urbanized, the rice value chain has transformed in specific ways, shifting from small, more localized rice mills to larger ones that are beginning to develop their own brands to sell differentiated rice in supermarkets, rather than wet markets. Minten et al. (2016) similarly describe changes in the teff value chain in Ethiopia near Addis Ababa. As urban consumers have become wealthier, they demand either teff flour or injera bread, meaning that new mills have developed to process teff more centrally and sell either to consumers or to bakeries to bake and then distribute injera. In both cases, entrepreneurs are apparently able to find sources of finance to develop these new midstream businesses.

The mandi system in India provides another nice example of market power, through government intervention, affecting the structure of agricultural markets (Chatterjee, 2023). In the mandi system, farmers are required to sell their product to government-licensed intermediaries within their own state; Chatterjee shows interstate trade restrictions and the fixed spatial distribution of markets reduces the prices farmers receive, and the licensing induces spatial market power among midstream license holders. If interstate trade restrictions were lifted, increased competition would increase farmgate prices (and output, according to his model). In this example, credit to farmers is likely affected, due to reduced returns to land and labor relative to what they would receive in the competitive counterfactual.

Second, many midstream activities require substantial lumpy fixed-cost investments for new entrants. Transporters may need trucks, cold storage requires cold rooms and power sources to cool them, and grain processing may require a mill. The specific requirements will depend on the value chain and the technology needed to perform the given task. In highly mechanized value chains these costs may be very high whereas in others, a midstream activity may only require additional labor and minimal additional financing. The more expensive and value-chain specific these items, the more likely that the level of competition at a specific value chain node will depend on access to finance. For example, investing in machines that can ultra-pasteurize milk for the sterile milk market requires value-chain-specific investment (Reardon and Minten, 2021). Lack of competition at a given node may also be the result of only certain actors being able to operate at a sufficient scale to achieve low marginal costs such as established distribution or supply networks and infrastructure (Williamson, 1995). Credit availability to these actors, through existing financial services and national policies, can therefore directly affect market concentration found at different nodes of agricultural value chains.

Third, requirements for export markets can directly affect value chain structure. Value chains serving international markets typically have a limited number of final buyers in the distribution phase, exporting either raw commodities or processed goods. The combination of quality standards required by importers in end markets and export licensing in exporting countries limits the number of buyers at the end of the chain, which can create market power (World Bank, 2007). However, in this context the presence of apex actors with access to high value markets and a lack of product can lead to potential finance opportunities.^10^

For example, Dries et al. (2009) summarize research in several countries in post-transition Central and Eastern Europe related to the development of a milk market for export to western Europe. To meet quality standards, farmers required both technical assistance and investment capital, while dairies required upgraded collection centers. Dairies that obtained foreign direct investment used that for collection centers, then co-signing bank loans with farmers to obtain additional high-quality milk and providing the required technical assistance.

Finally, market structure can affect the type of exchange relationships featured in different value chains. These relationships may include financial arrangements where larger actors help smaller ones, either up or downstream, by offering explicit or implicit credit, or contracts with lower risk exposure. The degree of formality in these relationships can vary greatly. In traditional value chains, most relationships are informal and transactional. As value chains modernize, relationships tend to become more formal, written as contracts with specific standards and conditions (Michler and Wu, 2020). In an extreme, when buyers find the transaction costs in transacting with sellers, they may vertically integrate specific parts of the value chain, which we discuss in more detail later.

## 4 The role of policy shaping the agricultural midstream

While the previous sections have outlined core functions and financial needs of midstream actors along with the implications of a set of value chain characteristics, we now turn to the way policy affects opportunities in the midstream. Given its importance for poverty reduction in most LMICs, governments have a long history of attempting to affect or increase domestic agricultural production. Here we take note of five different ways in which government policies or initiatives either directly or indirectly affect the financial landscape of agricultural midstream actors.

First, whether for political, strategic, or historical reasons, governments often attempt to promote specific value chains. Regardless of motivation, government involvement in specific value chains has implications for the structure of the midstream. Their interest typically focuses on primary staple crops (e.g. rice, maize) or key export crops. Much government involvement in staple markets has been reduced over time, either through privatization of state buyers that took place in Africa in the 1980s and 1990s (e.g. Kherallah et al., 2002) or through economic transition in formerly planned economies (Rozelle and Swinnen, 2004). MSME entry was hindered, at least initially, by factors such as sunk costs of entry and finance challenges (Barrett, 1997). Even though many governments have reduced or eliminated their involvement in staple output markets, some involvement continues, such as licensing of state level intermediaries in India (Chatterjee, 2023) or state-run firms that continue operating to either regulate staple prices or to attempt to ensure food security in the aggregate, such as the Food Reserve Agency in Zambia (Mason and Myers, 2013). At the extreme these restrictions can lead to market power that increases the spread between producer and consumer prices, as with the mandi system in India (Chatterjee, 2023).

Marketing boards or parastatals for key commodities constitute a second, more common, type of state involvement frequently motivated as a way to maintain foreign exchange balances (e.g. cocoa in Ghana, cotton in Mali, coffee in Uganda etc.).^11^ Marketing boards shape the opportunities available in the midstream for those commodities by playing roles such as licensing trader entry, enforcing quality standards, managing trading floors, and regulating disputes. An intermediary license to operate in any of these markets is valuable, as the limit on entry likely contributes to market power among intermediaries in the midstream (e.g. Fafchamps and Hill, 2008, Chatterjee, 2023).

Third, governments may establish commodity exchanges to formalize trade, ease price discovery, and help develop futures markets to limit price risks faced by market actors, at least for traded commodities. For example, the Ethiopia Commodity Exchange (ECX), established in 2008, represented a policy change affecting market function for export-oriented crops (Meijerink et al., 2014, Hernandez et al., 2017). Farmers who grow coffee as of late 2008 and sesame as of 2010 are required to sell their crops through primary transaction centers (PTCs), which at least theoretically have certified scales, market information boards, and local inspectors who certify the crops. Registered traders can then purchase crops from the PTCs and sell them to ECX regional warehouses. While warehouse receipts are issued for crops delivered to ECX warehouses, they are not tradeable by policy (Andersson et al., 2017), and high monthly fees for storage likely limits trading markets to well capitalized traders. Still, the ECX appears to have made coffee markets at least more efficient, as Andersson et al. (2017) find reduced price dispersion in coffee prices.

Financial sector regulation can also play a role in fostering, shaping, or constraining opportunities in the agricultural midstream. State-owned banks remain pervasive in many countries, which implies that financial policy may be tilted to ensure that such banks work both towards motives of profit and interests of policy makers (e.g. La Porta, Lopez-de-Silanes, and Shleifer, 2002). The interests of policy makers overseeing state-owned banks may not align with those in charge of fostering agricultural growth, hence affecting the midstream. That said, some of these banks have a mandate to work in rural areas and target the agricultural sector; when they exist, they can help provide agricultural credit, but they can also crowd out potential private sector investment. Moreover, state-owned banks may also not be structurally capable of working quickly to develop new products, may not have the existing incentives to do so, and may therefore require policy incentives to promote their innovation. Opportunities for entering financial markets may therefore be constrained, particularly if state-owned banks perceive threats to their business from new firms or offerings.

Finally, as we discuss in the following section, DFS are becoming an important part the financial landscape in many LMICs. National policy can have an important influence on the potential of DFS to satisfy the needs of actors in the agricultural midstream. Ideally, financial regulators ensure the legitimacy of market entrants and establish consumer protection safeguards, while not constraining innovation. Policies in the form of tax relief or subsidies can also be designed to promote entry, innovation, and potentially competition. With the right policy environment, firms within the financial sector can tailor products to the agricultural midstream. However, doing so may remain a challenge, as needs of agricultural midstream actors are not well understood.

## 5 Resolving midstream financial needs

In the previous sections, we provided a framework for thinking about the financial needs of midstream actors and their roles in modernizing value chains, as well as the government’s role in shaping those opportunities. In this section, we expand on the discussions about Table 1, Table 2, Table 3 to first consider how credit and insurance needs affect midstream actors. Next, we describe how vertical linkages or integration may be used by midstream actors to overcome these needs. Third, we briefly discuss how digital financial services may evolve to further serve the midstream.

### 5.1 Credit and insurance needs in the midstream

Table 1 of this paper detailed a list of short- and long-run liquidity challenges and financial risks faced by midstream actors. These needs vary by the type of value chain actor, the stage of modernization, and the logistical and market structure (Table 2, Table 3). Despite these differences, where available, well-designed financial instruments offered by banks and MFIs tailored to the needs of a specific midstream activity is likely the first-best financial solution for those actors. Formal credit insurance, transfer and payment services, savings vehicles, and market platforms all may at times serve these roles. However, information asymmetries, moral hazard, and limited liability impede the functioning of credit and insurance markets while idiosyncratic needs of diverse midstream actors may lead to poorly matched needs and available financial products. These unmet financial needs may manifest themselves in other ways along value chains such as when risk exposure and credit needs affect prices, forms of contracting, and vertical relationships between value chain actors.

Credit needs among midstream actors can vary widely. While large-scale private actors may be able to satisfy their own credit needs, by self-financing, accessing formal finance in the banking system, or by accessing international investment and financing (e.g., Van Campenhout et al., 2021), smaller and less formal MSMEs or individual actors in the midstream are less likely to have ready access to affordable credit. Considering the midstream activities detailed in Table 1, smaller actors working in transportation, trading, or processing are most likely to need liquidity in the short, though it is perhaps easiest to conceive of traders who purchase products directly from farmers as needing liquidity. These smaller actors are most likely to be present in traditional and transitional value chains (Table 2, Table 3), further highlighting the need for finance in these value chains. The broader literature in developing countries suggests that MSMEs across all sectors are frequently credit constrained and that there may be substantial returns to additional credit for many.^12^ However, most of these studies are based in urban samples, targeted towards non-agricultural activities, and thus may miss the particular credit needs and returns relevant to midstream agricultural SMEs. Finance for agricultural SMEs is distinct because of challenges related to the high costs of serving clients, the high and unique risks inherent in the market, particular repayment schedule and timing needs, and a client base that is less financially savvy and ready to make profitable investments (ISF Advisors, 2022).

One study directly considering financial needs of midstream agricultural MSMEs is Bergquist and Dinerstein (2020), who study market power among maize traders in Kenya. The authors find low take up of subsidized entry into trading. However, they also find that randomly induced entry into local markets does not affect local prices over the course of the study, attributing this non-response to collusion among traders. Expanding credit may therefore not be sufficient for reducing rents taken by large-scale actors in non-competitive value chain nodes.

In many LMICs, a general lack of collateral is a widespread challenge to credit provision. Warehouse receipt systems offer an alternative method of using crops to develop financial assets useful throughout the value chain. When farmers deliver crops for storage to a centralized warehouse, the crops are then graded and farmers receive a receipt for that crop. Those receipts become financial assets and can be used as collateral for bank loans throughout the value chain (Coulter and Onumah, 2002). Warehouse receipt systems work reasonably well in some places (e.g. Burkina Faso; see Le Cotty et al., 2019), but struggle in others, either because farmers lack trust in the receipt system in general (e.g. Casaburi et al., 2014) or because the benefit to farmers may outweigh the costs of transporting crops to the warehouses (de Brauw and Bulte 2021).

Exposure to uninsured risk also has the potential to deter or distort investments. Ideally, suitable, well priced insurance products would be available to directly cover the different sources of risk facing midstream actors. But it is questionable whether there is demand for insurance at actuarially fair rates. As a result, the lack of insurance can either lead to inflated prices to compensate for risk exposure and exclude would-be midstream entrants who are risk averse, or to different institutional arrangements. In Nicaragua, for example, Michelson et al. (2013) show that Walmart pays lower prices to local cooperatives than a local supermarket, La Colonia, but payments are less volatile. In other words, Walmart offers a type of price insurance for cooperatives relative to selling to their competitors, but it comes at the cost of lower farm returns on average.

### 5.2 Vertical linkages, vertical integration, and contract farming

Value chain actors do not operate in a vacuum. They are affected by upstream suppliers, downstream buyers, and also by how they engage with other actors at the same node as them. It is therefore important to consider these arrangements and how they may be affected by or themselves affect financial needs in the value chain.

Exposure to uninsured risk and unmet credit needs among value chain participants will affect both their upstream suppliers and downstream buyers. In the clearest case at the edge of the midstream, credit constraints and risk exposure of smallholders can directly impact midstream traders, particularly in traditional or transitional value chains. Technologies to improve yields or reduce exposure to shocks may exist, but credit constraints can prevent smallholders from adopting them. In a vicious circle, under-adoption of insurance to cover production risk may also lead to under investment and adoption of more productive or resilient technologies (Karlan et al., 2014, Cole et al., 2017, Lane, 2022). In either instance, this stunting of smallholder productivity translates into risk and reduced or unreliable supply for midstream actors. If unaddressed, these constraints may manifest themselves in negotiated contracts or informal agreements affecting prices or terms of exchange. In extreme cases, it may lead to market collapse where participation becomes unprofitable (Michler and Wu, 2020).

To increase the volume of their business, then, midstream actors have an incentive to help resolve or reduce financial constraints facing their suppliers or buyers. Contracting agreements are one mechanism by which value chain actors may account for these up or downstream financial constraints. Offering desirable contract features outside of pure quantity and price can help midstream actors expand their businesses and profit from processing, distributing, or exporting with a more stable supply of inputs. Guaranteeing a given price can reduce risk faced by one’s trading partners. Similarly, accepting delayed payments from borrowers or offering advance payments or providing inputs in-kind to suppliers may help relieve credit or liquidity constraints of value chain partners.

Depending upon the level of modernization of the value chains, contractual arrangements may be highly informal agreements made even implicitly between actors, or they may take the form of formal legal contracts (Michler and Wu, 2020). Casaburi and Reed (2022), in fact, exploit informal credit between traders and cacao producers to study how traders use credit as a component of compensation to compete for supply from upstream producers. Where contracts are costly to enter or impossible to enforce, midstream actors may instead find it more advantageous to go further and vertically integrate their operations. To date, most of the literature on formal contract farming has focused on impacts on smallholder farmers, leaving room for future work on its role for midstream actors.^13^

Agricultural value chain finance (AVCF) is a variant of contract farming that can play a role in helping finance reach smallholders and may benefit midstream actors under the right conditions (de Brauw and Swinnen 2023). AVCF leverages relationships between value chain actors to extend financial services (usually credit) to otherwise non-creditworthy smallholder farmers, improving the reliability of supply for midstream actors (Michler and Wu, 2020, Liverpool-Tasie et al., 2020, Barrett et al., 2022). A standard AVCF scheme allows a formal lender (e.g., a bank) to lend to a single midstream enterprise (e.g., a processor), which then lends to and buys commodities from individual farmers. The relationship between the enterprise and farmers can act as a substitute for more formal collateral provided by the farmers, as the enterprise can more effectively monitor and screen farmers than the bank, while the bank retains the ability to make a formal loan to an enterprise with a business easier for its loan officers to understand. These arrangements can involve credit, insurance, or a blend of both (e.g. Miller and da Silva, 2007).

As an example, van Campenhout et al. (2021) show that in Uganda, milk production for both domestic and export markets has recently rapidly expanded through an AVCF scheme. Milk production is concentrated in two geographic areas with two distinct value chain structures: one that is more modern, serving international markets and a second that is more traditional or transitional serving domestic regional markets. Innovation has been markedly faster in the more modern chain. Recently established milk collection centers in the faster growing area often provide credit, hygiene trainings, and aluminum milk cans to their clients (farmers). In most cases, credit flows from banks or Village Savings and Loan Associations to farmers, rather than directly from the cooperative established alongside the milk collection center. Moreover, a small number of traders who link farmers to collection centers have been able to obtain formal loans to provide working capital. In this context, the rapid expansion of such centers provides the competition necessary to keep any one actor from dominating returns. However, contracting with smallholders may be too costly—implicitly or explicitly—for midstream actors to justify. Reardon et al. (2021) suggest that “tied output-credit” arrangements between MSME wholesalers and farmers have been declining, attributing the shift to value chain modernization whereby improved infrastructure leads to competition among buyers.

When vertical contracting is too costly, a solution could instead be vertical integration—where a single company or actor incorporates several activities along the value chain. While most of the documented examples (including those cited above) consider links between midstream actors and producers, these dynamics may affect other value chain relationships. The consolidation of multiple value chain tasks is typically undertaken by a large actor with sufficient capital to make the necessary investments to straddle multiple nodes of the value chain, and is a feature of modern value chains. For example, pineapple exporters in Ghana typically manage plantations for much of their product need, buying additional surplus from local smallholders when demand is particularly high (Suzuki et al., 2011). For export firms or large processors, when transaction costs are too high to deal with large numbers of suppliers, if possible they will try to vertically integrate value chain nodes (e.g. Beghin et al., 2015). In Africa, there have been several efforts to vertically integrate farmers for export markets such as fruits, cut flowers and vegetables (Feyaerts et al., 2019). As vertical integration occurs, the midstream absorbs production which may crowd out smallholder farmers and create opportunities for hired rural employment (Maertens and Swinnen, 2012, Van den Broeck et al., 2017).

As a nice example of an apex actor using access to resources to take on more midstream activities, tea plantations frequently do their own processing and even distribution once they reach a reasonable size (Munyambonera et al., 2014). While we typically think of vertical integration as being driven by larger scale actors, small scale farmers in essence vertically integrate some midstream activities as they expand into value added activities or even storage, although evidence suggests that credit constraints can create a meaningful barrier to these efforts (Burke et al., 2019).

### 5.3 Digital financial services

The recent and rapid expansion of mobile phone availability and the accompanying emergence of DFS are creating promising new forms of financial services.^14^ There are many reasons why DFS may have the potential to expand financial offerings in the agricultural sector. Low marginal costs of service expansion may allow digital services to penetrate markets where conventional reliance on brick-and-mortar presence is financially infeasible. In turn, the services can result in lower usage costs or better terms for adopters when they reach scale, expanding financial access (e.g. Suri et al., 2021). In addition to low service fees, using DFS can reduce search costs and payment processing delays that often accompany formal services. Digital platforms also have the potential to take more innovative forms, offering more adaptable and tailored services where standardized products and existing services have not been well matched to users’ needs.^15^ Here, we note several ways in which we believe that DFS may offer particular benefits to agricultural midstream actors and have the potential to resolve some of the financial challenges discussed above.

First, the previous section discussed how the midstream is affected by financial constraints of upstream smallholders. If DFS could begin to relieve financial constraints among some smallholders and induce either a production increase or a change in production patterns, it could substantially affect the midstream, since midstream actors would need to absorb those changes. It could also reduce the prevalence of tied credit arrangements between some midstream actors and smallholders, and help catalyze a shift from traditional to transitional value chains. Relieving smallholders’ credit or insurance constraints would reduce an existing distortion in the ways that value chain actors interact.

Second, mobile money and digital transactions may also have considerable impacts on midstream actors, especially for firms that make frequent payments to a large number of partners. There is considerable untapped potential for digital payments in agriculture, where about 235 million unbanked farmers and actors receive agricultural payments in cash, and 59% own a mobile phone (Demirguç-Kunt et al., 2018). Digital transfer systems have the potential to reduce transaction costs and create greater efficiency for transacting partners (Suri et al., 2023). They may additionally lower exposure to theft or loss that would result from holding large amounts of money needed for cash-based transactions. Midstream exporters or processors who source their inputs from a recurrent set of suppliers, with high numbers of transactions, are likely to have the greatest returns to lowering transaction costs and (depending on the accompanying service fees) adopting digital payment systems (GSMA, 2018). Going even further, digital transactions can also generate verifiable records of past activities that can increase accountability and traceability of past transactions, improving overall value of a given value chain as well as creating opportunities for digital credit scoring and expansion of credit opportunities (Björkegren and Grissen, 2020, Aker and Cariolle, 2023).

A third promising area of technological innovation has been the proliferation of mobile or online marketplaces for agricultural commodities, which have the potential to catalyze value chain modernization (ISF Advisors, 2021). These platforms are meant to facilitate transactions either between farmers and traders or between aggregators and processors, and sometimes link farmers directly to retailers, such as Twiga Foods in Kenya (Chege et al., 2023). Online marketplaces can also link with other services, including credit or advisory services (Abate et al., 2023). Evidence about their effectiveness is mixed. Bergquist, McIntosh, and Startz (2023) study the impacts of an SMS based marketplace to reduce search costs in Ugandan grain markets, finding reductions in price dispersion between relative surplus and deficit markets. However, consistent with economies of scale, marketplace adoption was limited to large-scale wholesalers. Tsan et al. (2019) suggest that the technical capabilities of online marketplaces exceed necessary conditions for their viability, such as digital literacy. Further, many platforms that exist to date remain unprofitable (ISF Advisors, 2021), perhaps because they struggle to reach large enough scale. Iavocone and McKenzie (2022), who study a fruit and vegetable delivery service in Colombia, find that the struggle to reach scale led to service closure, despite a clear market failure among vendors in obtaining products to sell. From a policy perspective, unifying digital platforms could help platforms reach scale, as has been attempted in Karnataka, India (Levi et al., 2020).^16^

The broad menu of evolving digital services has potential to catalyze the development of many agricultural value chains. However, technical capabilities seem to be ahead of the necessary environmental conditions such as human capital and digital literacy in many circumstances (Tsan et al., 2019). As such, midstream actors may be an attractive entry point for such services as they are likely to have higher digital literacy, access, and connectivity (Denyes et al., 2019). Further, outside of a few leading countries (e.g. India and Kenya), private sector investments remain low and successful products have been mostly donor driven. Where tech start-up firms are involved, reach is often low and financial sustainability has not been achieved (Tsan et al., 2019). However, as value chains move from traditional to modern, midstream actors in value chains close to or in the transitional phase are poised to benefit from such products. The features of these chains, such as a longer midstream, diverse end markets, more formal contracting, greater credit demands, and possibly higher exposure to price risk, suggests that digital solutions could have substantial impact. At the same time, the enabling environment is evolving, suggesting that these actors may also be ready to take advantage of the right service.

## 6 Conclusion

In recent decades, agricultural value chains in many LMICs have undergone a rapid transformation. Their reorganization has implications for how income and welfare are shared throughout the value chain. Yet as this paper argues, the midstream of those value chains has received limited research attention and the role that finance plays in their businesses is understudied. The vast majority of midstream businesses are MSMEs and in LMICs they are known, in general, to lack access to capital. The unique features of agriculture, including its spatial dispersion and seasonality, suggest that agricultural MSMEs in the midstream may face even more difficult challenges accessing capital and other suitable financial services. These challenges may in turn hinder entry into this sector and contribute to lack of competitiveness at certain nodes of value chains. Further, although midstream actors may have greater access to finance than upstream smallholders, they still rely on these upstream actors for their inputs. Where smallholders’ financial needs are not being met, a lack of smallholder access to finance can reduce midstream actors’ supply and profitability.

This paper has documented the need for further research into financial constraints for midstream actors. We highlight some of those gaps here. First, there is a broad need to better understand how midstream actors finance their activities, particularly with regards to working capital and any capital expenses they require. In general, little is known about the ability of different types of midstream actors to access finance, the constraints they face in doing so, and whether financial constraints hinder firm entry. Second, more research on the role that risk plays in shaping outcomes among MSMEs working in the midstream would help build an understanding of behaviors that such firms currently undertake to mitigate risk, and to design insurance or other products to help alleviate constraints posed by uninsured risk. Third, more research on vertical linkages within value chains could lead to an improved understanding of how those linkages affect outcomes among different types of midstream actors. A related question is how those outcomes differ either by agricultural product, or the level of government involvement within chains. Last, given the growth of digital financial services in recent years, further research could lead to a better understanding of appropriate roles for those services to help grow income among midstream actors. Even while our attention in this article is primarily on the midstream actors, we maintain the importance of allocating attention to the upstream impacts of these evolving changes in the midstream on smallholders, to ensure that smallholder income is not reduced by the emergence of such services.

## Declaration of Competing Interest

The authors declare that they have no known competing financial interests or personal relationships that could have appeared to influence the work reported in this paper.

## Footnotes

1 Whether improved performance of smallholders can both drive poverty reduction and long run economic growth is a disputed claim within the literature. At some point in the development process, some farm consolidation is necessary to reduce costs of marketing surplus through value chains (Collier and Dercon, 2014). Dercon and Gollin (2014) further note that the sheer number of smallholders within a country does not guarantee investments targeting them will have high returns in terms of poverty reduction or growth; agricultural productivity gains may also be easier to attain with larger farms. Nonetheless, an important constraint can be the size and development level of markets in which farmers sell their surplus.

2 These numbers are unweighted totals of the pooled data. Ambler et al. (2022a) discusses further details about the approach taken to estimating these proportions.

3 Ambler et al. (2022b) includes for a detailed list of the surveys reviewed and their contents.

4 We also acknowledge that index insurance for smallholder farmers still faces substantial challenges in getting farmers to value and be willing to take it up at sustainable, non-heavily subsidized market rates.

5 Very large business may find other ways of hedging on these prices and mitigating risk exposure, but MSMEs in the midstream of most transitional value chains are unlikely to have this capacity.

6 For example, products that spoil easily or require stricter quality controls may have a different structure than chains for products that are more stable or require less regulation.

7 See Reardon et al. (2021), Reardon and Minten (2021), and Barrett et al. (2022) for rich discussions on and examples of the progression of agricultural value chain modernization.

8 BTCA (2020) quantifies the inefficiency in Ghana’s Cocoa sector related to the risks and costs of high cash needs in a geographically disperse value chain.

9 On the production side of the value chain, staple crops are often widespread, while products requiring more specialized agroecological conditions will be concentrated in areas with those conditions. Varying agroecological conditions also have implications for the types of financial services that midstream actors require.

10 Value chains with quality standards are also good opportunities to implement digital traceability systems to track and ensure quality. Well-functioning traceability systems can facilitate finance access. However, barriers such as smart phone access and poor data entry make traceability systems difficult to implement well (Powell et al., 2022).

11 Marketing boards or value chains for many key commodities were liberalized with structural adjustment in the 80s and 90s, similar to staple crops. However, there are some examples that only underwent partial liberalization; e.g. cocoa in Ghana (Kolavalli and Vigneri, 2017).

12 De Mel et al. (2008) use randomized grants for microenterprises in Sri Lanka and find average real returns to capital of 4.6% to 5.3% per month, above the actual market interest rates; these returns are lasting (De Mel et al., 2012). Fafchamps et al. (2014) give cash and in-kind grants to firms in Ghana, finding the in-kind grants lead to higher returns particularly female entrepreneurs, suggesting self-control problems with cash. Blattman et al. (2014) found grants to youth led to 38 percent gains in income; nine years later those impacts had dissipated (Blattman et al., 2020). McKenzie (2017) further shows high returns gained by winners of a randomized business plan competition in Nigeria.

13 Recent literature reviews on contract farming include Otsuka et al. (2016), Minot and Sawyer (2016), and Bellemare and Bloem (2018).

14 See Abate et al (2023) for an example of a recent summary of emerging digital services of all types and their implications for the agricultural sector in sub-Saharan Africa.

15 Aker and Cariolle (2023) review the performance of mobile phones in Africa, and find that while they can reduce search costs, improve access to information, and help households and businesses smooth consumption, their effectiveness in improving overall welfare is more mixed and depends upon other factors.

16 Digital credit and insurance services are other DFS offerings, largely for smallholders, that hold some promise for midstream actors. For example, digital data sources, including the type of transaction histories generated from the use of digital payment systems may be used for digital credit scoring and assessments of creditworthiness (Björkegren and Grissen, 2020). However, few if any of these offerings are tailored to midstream actors.

## References

[b0005] Abate G.T., Bernard T., de Janvry A., Sadoulet E., Trachtman C. (2021). Introducing quality certification in staple food markets in Sub-Saharan Africa: four conditions for successful implementation. Food Policy.

[b0010] Abate G.T., Abay K.A., Chamberlin J., Kassim Y., Spielman D.J., Tabe-Ojong M.P. (2023). Digital tools and agricultural market transformation in Africa: why are they not at scale yet, and what will it take to get there?. Food Policy.

[b0015] AGRA, 2019. Africa Agriculture Status Report: The hidden middle: A quiet revolution in the private sector driving agricultural transformation (issue 7). Nairobi, Kenya: Alliance for a Green Revolution in Africa (AGRA). https://agra.org/wp-content/uploads/2019/09/AASR2019-The-Hidden-Middleweb.pdf.

[b0020] Aker J.C., Cariolle J. (2023).

[b0025] Ambler K., de Brauw A., Herskowitz S., Pulido C. (2022). Financial Access of Midstream Firms in Africa: Evidence From the LSMS-ISA and World Bank Enterprise Surveys.

[b0030] Ambler K., de Brauw A., Herskowitz S., Pulido C. (2022). Value chain surveys: What do they cover, and how well?. IFPRI Project Note.

[b0035] Andersson C., Bezabih M., Mannberg A. (2017). The Ethiopian Commodity Exchange and spatial price dispersion. Food Policy.

[b0040] Barrett C.B. (1997). Food Marketing Liberalization and Trader Entry: Evidence from Madagascar. World Dev..

[b0045] Barrett C.B., Reardon T., Swinnen J., Zilberman D. (2022). Agri-food value chain revolutions in low-and middle-income countries. J. Econ. Lit..

[b0050] Beghin J., Maertens M., Swinnen J.F.M. (2015). Nontariff measures and standards in trade and global value chains. Ann. Rev. Resour. Econ..

[b0055] Bellemare M.F., Bloem J.R. (2018). Does contract farming improve welfare? A review. World Dev..

[b0060] Bellemare, M.F., Bloem, J.R., Lim, S.,2022. Producers, Consumers, and Value Chains in Low-and Middle-Income Countries*.* In: Barrett, C., Just, D. (Eds.) Handbook of Agricultural Economics, vol. 6, Elsevier, Amsterdam, pp. 4933-4996. (Chapter 89).

[b0065] Bergquist L.F., Dinerstein M. (2020). Competition and entry in agricultural markets: experimental evidence from Kenya. Am. Econ. Rev..

[b0070] Bergquist, L.F., McIntosh, C., Startz, M., 2023. Search Costs, Intermediation, and Trade: Experimental Evidence from Ugandan Agricultural Markets. In: Paper presented at 2023 NBER Summer Institute Development Economics Meeting. https://conference.nber.org/conf_papers/f189058.pdf.

[b0075] Björkegren D., Grissen D. (2020). Behavior revealed in mobile phone usage predicts credit repayment. World Bank Econ. Rev..

[b0080] Blattman C., Fiala N., Martinez S. (2014). Generating skilled self-employment in developing countries: Experimental evidence from Uganda. Q. J. Econ..

[b0085] Blattman C., Fiala N., Martinez S. (2020). The long-term impacts of grants on poverty: nine-year evidence from Uganda's youth opportunities program. Am. Econ. Rev.: Insights.

[b0090] Better Than Cash Alliance (BTCA), 2016. Responsible Digital Payments Guidelines. Better Than Cash Alliance Report, New York. https://www.betterthancash.org/explore-resources/responsible-digital-payments-guidelines.

[b0095] Better Than Cash Alliance (BTCA), 2020. The Hidden Costs of Cash to Ghana’s Cocoa Sector. Better Than Cash Alliance Report, New York. https://www.betterthancash.org/explore-resources/the-hidden-costs-of-cash-to-ghanas-cocoa-sectors.

[b0100] Burke M., Bergquist L.F., Miguel E. (2019). Sell low and buy high: arbitrage and local price effects in Kenyan markets. Q. J. Econ..

[b0105] Casaburi, L., Glennerster, R., Suri, T., Kamara, S., 2014. Providing collateral and improving product market access for smallholder farmers: a randomised evaluation of inventory credit in Sierra Leone. 3ie Impact Evaluation Report 14. https://www.3ieimpact.org/evidence-hub/publications/impact-evaluations/providing-collateral-and-improving-product-market.

[b0110] Casaburi L., Reed T. (2022). Using individual-level randomized treatment to learn about market structure. Am. Econ. J. Appl. Econ..

[b0115] Chatterjee S. (2023). Market Power and Spatial Competition in Rural India. Q. J. Econ..

[b0120] Chege, C., Onyango, K., Lundy, M., Kabach, J., 2023. Small and medium enterprises (MSMEs) disrupt food systems to deliver healthy diets to urban consumers: Twiga case study, Nairobi Kenya. Sustainable Healthy Diets through Food Systems Transformation working paper. https://hdl.handle.net/10568/130733.

[b0125] Cole S., Giné X., Vickery J. (2017). How does risk management influence production decisions? Evidence from a field experiment. Rev. Financ. Stud..

[b0130] Collier P., Dercon S. (2014). African agriculture in 50 years: smallholders in a rapidly changing world?. World Dev..

[b0135] Coulter J., Onomah G. (2002). The role of warehouse receipt systems in enhanced commodity marketing and rural livelihoods in Africa. Food Policy.

[b0140] de Brauw, A, Swinnen, J., 2023. Building Inclusive Value Chains for Smallholders: The Role of Finance. In: Hartarska, V., Cull, R. (Eds.) Handbook of Microfinance, Inclusion, and Development, Edward Elgar, London, pp. 181-193. (Chapter 11).

[b0145] de Brauw A., Bulte E. (2021).

[b0150] De Mel S., McKenzie D., Woodruff C. (2008). Returns to capital in microenterprises: evidence from a field experiment. Q. J. Econ..

[b0155] De Mel S., McKenzie D., Woodruff C. (2012). One-time transfers of cash or capital have long-lasting effects on microenterprises in Sri Lanka. Science.

[b0160] Demirguc-Kunt A., Klapper L., Singer D., Ansar S., Hess J. (2018).

[b0165] Denyes L., Lesher N., Martinez M., McNutt H., Nakhai M. (2019).

[b0170] Dercon S., Gollin D. (2014). Agriculture in African development: theories and strategies. Ann. Rev. Resour. Econ..

[b0175] Dolislager M., Reardon T., Arslan A., Fox L., Liverpool-Tasie S., Sauer C., Tschirley D.L. (2021). Youth and adult agrifood system employment in developing regions: Rural (peri-urban to hinterland) vs. urban. J. Dev. Stud..

[b0180] Dorosh P., Thurlow J. (2018). Beyond Agriculture *Versus* Non-Agriculture: Decomposing Sectoral Growth-Poverty Linkages in Five Countries. World Dev..

[b0185] Dries L., Germenji E., Noev N., Swinnen J. (2009). Farmers, vertical coordination, and the restructuring of dairy supply chains in Central and Eastern Europe. World Dev..

[b0190] Fafchamps M. (2003).

[b0195] Fafchamps M., Hill R.V. (2008). Price transmission and trader entry in domestic commodity markets. Econ. Dev. Cult. Chang..

[b0200] Fafchamps M., McKenzie D., Quinn S., Woodruff C. (2014). Microenterprise growth and the flypaper effect: evidence from a randomized experiment in Ghana. J. Dev. Econ..

[b0205] Fafchamps M., Minten B. (2001). Property rights in a flea market economy. Econ. Dev. Cult. Chang..

[b0210] Fafchamps M., Minten B. (2002). Returns to social network capital among traders. Oxf. Econ. Pap..

[b0215] Fayaerts H., van den Broeck G., Maertens M. (2020). Global and local food value chains in Africa: a review. Agric. Econ..

[b0220] GSMA, 2018. The GSMA mAgri value chain assessment tool: A framework for prioritising digital interventions in the agricultural last mile. GSMA Report, London. https://www.gsma.com/mobilefordevelopment/resources/magri_value_chain_tool/.

[b0225] Hernandez R., Belton B., Reardon T., Hu C., Zhang X., Ahmed A. (2018). The “quiet revolution” in the aquaculture value chain in Bangladesh. Aquaculture.

[b0230] Hernandez M., Rashid S., Lemma S., Kuma T. (2017). Market institutions and price relationships: the case of coffee in the ethiopian commodity exchange. Am. J. Agric. Econ..

[b0235] Iacovone L., McKenzie D. (2022). Shortening supply chains: experimental evidence from fruit and vegetable vendors in Bogota. Econ. Dev. Cult. Chang..

[b0240] ISF Advisors, 2021. Agricultural “platforms” in a digital era: Defining the landscape. ISF Advisors Report, Washington DC. https://isfadvisors.org/wp-content/uploads/2021/03/ISF_RAFLL_Agricultural_Platforms_Report.pdf.

[b0245] ISF Advisors, 2022. The state of the agri-SME sector – Bridging the finance gap. Commercial Agriculture for Smallholders and Agribusiness (CASA) Programme Report, Washington DC. https://www.casaprogramme.com/wp-content/uploads/2022/03/the-state-of-the-agri-sme-sector-bridging-the-finance-gap.pdf.

[b0250] Johnston B.F., Mellor J.W. (1961). The role of agriculture in economic development. Am. Econ. Rev..

[b0255] Karlan D., Osei R., Osei-Akoto I., Udry C. (2014). Agricultural decisions after relaxing credit and risk constraints. Q. J. Econ..

[b0260] Kherallah M., Delgado C.L., Gabre-Madhin E.Z., Minot N., Johnson M. (2002).

[b0265] Kolavalli, S., Vigneri, M., 2017. The Cocoa Coast: The Board-Managed Cocoa Sector in Ghana. Washington, DC: International Food Policy Research Institute. 10.2499/9780896292680.

[b0270] La Porta R., Lopez-De-Silanes F., Shleifer A. (2002). Government ownership of banks. J. Financ..

[b0275] Lane, G., 2022. Adapting to Floods with Guaranteed Credit: Evidence from Bangladesh. Working Paper, Harris School of Public Policy, University of Chicago. https://www.gregoryvlane.com/pdfs/Lane_Floods.pdf.

[b0280] Le Cotty T., Maître D’Hôtel E., Soubeyran R., Subervie J. (2019). Inventory credit as a commitment device to save grain until the hunger season. Am. J. Agric. Econ..

[b0285] Levi R., Rajan M., Singhvi S., Zheng Y. (2020). The impact of unifying agricultural wholesale markets on prices and farmers’ profitability. Proc. Natl. Acad. Sci..

[b0290] Liverpool-Tasie L.S.O., Wineman A., Young S., Tambo J., Vargas C., Reardon T., Adjognon G.S., Porciello J., Gathoni N., Bizikova L., Galie A., Celestin A. (2020). A scoping review of market links between value chain actors and small-scale producers in developing regions. Nat. Sustainability.

[b0295] Maertens M., Swinnen J.F.M. (2012). Gender and modern supply chains in developing countries. J. Dev. Stud..

[b0300] Mason N.M., Myers R.J. (2013). The effects of the food reserve agency on maize market prices in Zambia. Agric. Econ..

[b0305] McKenzie D. (2017). Identifying and spurring high-growth entrepreneurship: experimental evidence from a business plan competition. Am. Econ. Rev..

[b0310] Meijerink G., Bulte E., Alemu D. (2014). Formal institutions and social capital in value chains: the case of the Ethiopian commodity exchange. Food Policy.

[b0315] Michelson H., Reardon T., Perez F. (2013). Small farmers and big retail: trade-offs of supplying supermarkets in Nicaragua. World Dev..

[b0320] Michler J.D., Wu S.Y. (2020). Relational contracts in agriculture: theory and evidence. Ann. Rev. Resour. Econ..

[b0325] Miller C., da Silva C. (2007). Value chain financing in agriculture. Enterprise Dev. Microfinance.

[b0330] Minot, N., Sawyer, B., 2016. Contract farming in developing countries: Theory, practice, and policy implications. In: Devaux, A., Torero, M., Donovan, J., Horton, D.E. (Eds.) Innovation for Inclusive Value-Chain Development: Successes and Challenges, International Food Policy Research Institute, Washington, DC, pp. 127-158. (Chapter 4).

[b0335] Minten B., Randrianarison L., Swinnen J.F.M. (2009). Global retail chains and poor farmers: evidence from Madagascar. World Dev..

[b0340] Minten B., Stifel D., Tamru S. (2014). Structural transformation of cereal markets in Ethiopia. J. Dev. Stud..

[b0345] Minten B., Tamru S., Enigda E., Kuma T. (2016). Transforming Staple food value chains in Africa: the case of Teff in Ethiopia. J. Dev. Stud..

[b0350] Munyambonera, E., Lakuma, P.C., Madina, G., 2014. Uganda’s Tea Sub-Sector: A Comparative Review of Trends, Challenges and Coordination Failures. Economic Policy Research Centre Research Series no. 119.

[b0355] Otsuka K., Nakano Y., Takahashi K. (2016). Contract farming in developed and developing countries. Ann. Rev. Resour. Econ..

[b0360] Powell W., Foth M., Cao S., Natanelov V. (2022). Garbage in garbage out: The precarious link between IoT and blockchain in food supply chains. J. Ind. Inf. Integr..

[b0365] Reardon T. (2015). The hidden middle: the quiet revolution in the midstream of agrifood value chains in developing countries. Oxf. Rev. Econ. Policy.

[b0370] Reardon, T., Minten, B., 2021. Food value chain transformation in developing regions. In: Otsuka, K., Fan, S. (Eds.) Agricultural Development: New perspectives in a changing world. Washington, DC: International Food Policy Research Institute, pp. 397-458. 10.2499/9780896293830_12 (Chapter 12).

[b0375] Reardon T., Chen K., Minten B., Adriano L. (2012).

[b0380] Reardon T., Chen K.Z., Minten B., Adriano L., Dao T.A., Wang J., Das Gupta S. (2014). The quiet revolution in Asia's rice value chains. Ann. N. Y. Acad. Sci..

[b0385] Reardon T., Liverpool-Tasie L.S.O., Minten B. (2021). Quiet Revolution by SMEs in the midstream of value chains in developing regions: wholesale markets, wholesalers, logistics, and processing. Food Security.

[b0390] Rozelle S., Swinnen J. (2004). Success and failure of reform: insights from the transition of agriculture. J. Econ. Lit..

[b0395] Sitko N.J., Jayne T.S. (2014). Exploitative briefcase businessmen, parasites, and other myths and legends: assembly traders and the performance of maize markets in eastern and southern Africa. World Dev..

[b0400] Suri T., Bharadwaj P., Jack W. (2021). Fintech and household resilience to shocks: Evidence from digital loans in Kenya. J. Dev. Econ..

[b0405] Suri T., Aker J., Batista C., Callen M., Ghani T., Jack W., Klapper L., Riley E., Schaner S., Sukhtankar S. (2023). Mobile money. VoxDevLit.

[b0410] Suzuki A., Jarvis L.S., Sexton R. (2011). Partial vertical integration, risk shifting, and product rejection in the high-value export supply chain: the Ghana pineapple sector. World Dev..

[b0415] Swinnen J., Kuijpers R. (2019). Value chain innovations for technology transfer in developing and emerging economies: conceptual issues, typology, and policy implications. Food Policy.

[b0420] Timmer, C.P., 2002. Agriculture and economic development. In: Gardner, B., Rausser, G. (Eds.) Handbook of Agricultural Economics, vol. *2*, part A, Elsevier, Amsterdam, pp. 1487-1546. 10.1016/S1574-0072(02)10011-9 (Chapter 29).

[b0425] Tsan M., Totapally S., Hailu M., Addom B.K. (2019).

[b0430] United Nations, 2022. The Sustainable Development Goals Report. New York: United Nations. https://unstats.un.org/sdgs/report/2022/.

[b0435] Van Campenhout B., Minten B., Swinnen J.F. (2021). Leading the way–foreign direct investment and dairy value chain upgrading in Uganda. Agric. Econ..

[b0440] Van den Broeck G., Swinnen J., Maertens M. (2017). Global value chains, large-scale farming, and poverty: Long-term effects in Senegal. Food Policy.

[b0445] Williamson O.E. (1995). Hierarchies, markets and power in the economy: an economic perspective. Ind. Corp. Chang..

[b0450] World Bank, 2007. World Development Report 2008: Agriculture for Development, The World Bank, Washington, DC. http://hdl.handle.net/10986/5990.

[b0455] Yeboah F.K., Jayne T.S. (2018). Africa’s evolving employment trends. J. Dev. Stud..

[b0460] Yi J., Meemken E.M., Mazariegos-Anastassiou V., Liu J., Kim E., Gómez M.I., Canning P., Barrett C.B. (2021). Post-farmgate food value chains make up most of consumer food expenditures globally. Nature Food.

